# Anosmic flies: what *Orco* silencing does to olive fruit flies

**DOI:** 10.1186/s12863-020-00937-0

**Published:** 2020-12-18

**Authors:** Konstantina T. Tsoumani, Alexandros Belavilas-Trovas, Maria-Eleni Gregoriou, Kostas D. Mathiopoulos

**Affiliations:** Department of Biochemistry and Biotechnology, University of Thessaly, Biopolis, 41500 Larissa, Greece

**Keywords:** RNAi, Olfactory co-receptor, Oviposition, Mating disruption, Reproductive behavior

## Abstract

**Background:**

The olive fruit fly (*Bactrocera oleae*) is the most destructive pest of the olive cultivation worldwide causing significant production losses and olive fruit impoverishment, as its larvae feed exclusively on the olive fruit. Reproductive and sexual behavior, as well as host-plant recognition of the fly, are highly dependent on its chemosensory system. Therefore, exploring the role of genes that play a critical role in olfaction, could reveal potential molecular targets that determine species-specific features on chemical communication and could be used to impair sexual behavior.

**Results:**

In this study we identified the gene that encodes the conserved olfactory co-receptor *Orco* (Odorant receptor co-receptor), which interacts with all divergent insect odorant receptors, and investigated how disruption of its expression affects chemoreception. We initially searched the expression profile of *Bo-Orco* in both sexes during sexual maturation, as well as pre- and post-mating communication by relative quantitative real time PCR (qRT-PCR) analysis suggesting that *Bo-Orco* was abundantly expressed in sexually mature adults.

We further investigated the functional role of *Bo-Orco* in mating and oviposition behavior via transient gene silencing that was performed through in vivo dsRNA hemolymph injections in sexually mature flies 7 days after eclosion. *Orco*-knockdown phenotypes in both sexes showed reduced copulation rates in mating competitiveness tests, possibly through impaired olfactory-mediated detection of sex pheromone. In addition, oviposition was significantly inhibited in dsRNA-Orco injected females in a post-mating behavior test.

**Conclusions:**

Our results demonstrate that *Orco* plays a crucial role in the reproductive behavior of the olive fruit fly, since pre- and post-mating processes were affected. This is the first report in the olive fruit fly that links the chemosensory pathway with the mating behavior and the reproductive potential at a molecular basis, rendering this gene a potential target for the improvement of the olive fruit fly population control techniques.

## Background

The olive fruit fly (*Bactrocera oleae*) is the most destructive pest of the olive cultivation worldwide causing significant losses to the production and olive fruit impoverishment. The caused damage results mainly from the oviposition stings onto the olive fruits by the female insects while laying their eggs, as well as from the pulp destruction by its developing monophagous larvae [1, 2]. These detrimental consequences are associated with reproductive success (mating and oviposition) and effective host-seeking. For both of these developmental and reproductive processes to occur, the flies exploit chemosensory signals or cues to respond to biotic and abiotic environmental factors and also adapt to their own physiological states [3]. For instance, insects should be reproductive mature in order to respond to sexual signals under the appropriate environmental conditions necessary for their life history traits.

A multiple level sensory system modulates the peripheral reception of semiochemicals and central nervous system processing, which is ultimately translated into odor-guided behavior. The plasticity of the respective behavioral responses to given chemical stimuli is well regulated by the olfactory system.

The main olfactory organs, the antennae and the maxillary palps, are covered by sensilla which contain the olfactory receptor neurons (ORNs). Odorant molecules penetrate insect’s sensilla pores, and then are transferred by odorant binding proteins (OBPs) to the ORN membrane, where olfactory receptors (ORs) are located. Activation of the ORs will ultimately lead to signal transduction [4]. ORs interact with olfactory receptor co-receptor (Orco), which is evolutionary conserved among insect species [5]. Orco is co-expressed with ORs to form heterodimers that function as ligand-gated ion channels, as proposed by several studies [6, 7]. Recently Butterwick and colleagues [8] provided structural insights to the real structure of Orco by Cryo-electron microscopy, demonstrating its novel homotetrameric architecture and revealing in more detail possible structure–function relation.

Such advances on both molecular and cellular insight have greatly contributed towards the development of novel control strategies based on the manipulation of olfactory-guided behaviors particularly implicated in mate seeking and oviposition.

In *B. oleae*, although significant economic importance has been attributed to this pest, the factors driving its chemosensory communication at a molecular level are poorly studied so far, hampering the opportunities to disrupt its reproductive process. Morphological [9], electrophysiological [10, 11], as well as behavioral ecology studies [12–14] have provided evidence mainly regarding the insect’s responses to volatile molecules, including general plant odors and pheromones. The latter have been extensively studied for several decades demonstrating that, in contrast with the majority of Tephritidae, the main sexual pheromone in *B. oleae* is emitted by the female [15]. It consists of a four component mixture with 1,7-dioxaspiro [5.5] undecane, also called olean*,* as its main component [16, 17]. Furthermore, recent studies have demonstrated that males are also able to produce a compound that selectively attracts females, named (Z)-9-tricosene (muscalure), which is considered as the male pheromone [18, 19].

Therefore, exploring the role of genes that play a critical role in olfaction, could reveal potential molecular targets that determine species-specific features on chemical communication and could be used to impair sexual behavior. Orco coreceptor, based on its conserved role in insect survival mediating olfactory responses, could, in principle, be a suitable and effective target for manipulating insect behavior.

In this work we identified the *B. oleae* olfactory co-receptor (*Bo-Orco*) and examined the differential expression of this gene between males and females under different physiological conditions. We further proceeded with various functional analyses via RNA interference (RNAi) gene silencing, a method that was also successfully implemented in *B. oleae* embryos by microinjection [20] but also in adults via dsRNA feeding [21]. We performed transient knockdown in adult flies in order to assess Orco’s possible role in mating and oviposition behavior of the fly. This was the first attempt to examine the olfactory system of *B. oleae* at a molecular level, aiming at the identification of putative molecular targets for *B. oleae* control that could be proven useful in the development of new, more efficient pest control strategies.

## Results & discussion

### Sequence analysis of *Bo-Orco*

**Fig. 1:**
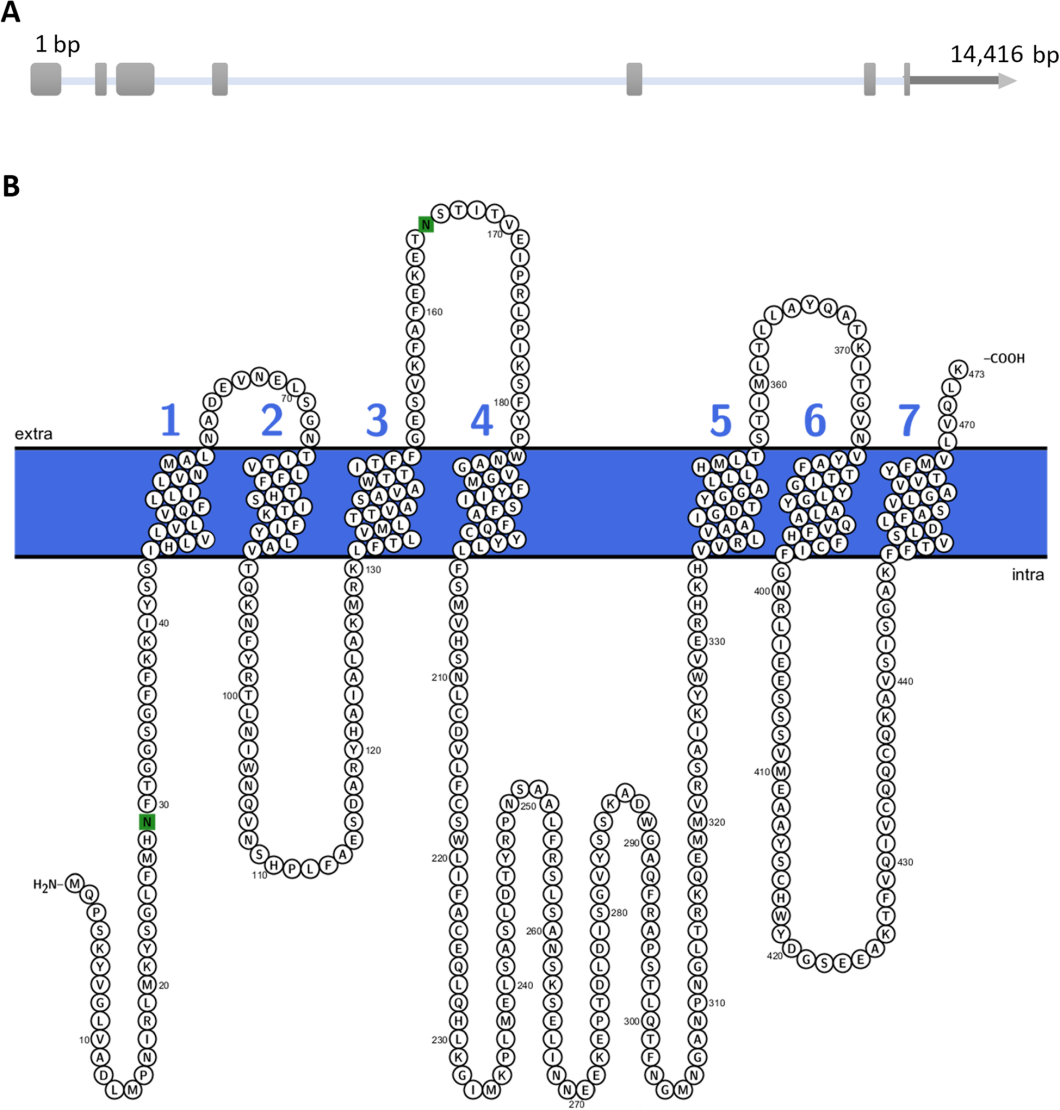
The *Bo-Orco* gene and protein structure. **a** Schematic representation of the genomic organization of the *Orco* gene. Grey boxes correspond to the exons and the grey line to the UTR. **b** Protter plot showing the secondary structure of Bo-Orco. The transmembrane topology of Bo-Orco was predicted using TMHMM. Transmembrane domains are indicated by colored numbers 1–7

The *Bo-Orco* gene (GenBank acc. no. XM_014236978.1) has been identified by homology Blastp searches against the NCBI non-redundant (nr) protein database of *Bactrocera oleae* using the *Drosophila melanogaster* orthologue as a query. The gene structure (JAMg_model_4806.1) was identified by manual curation at the i5k Workspace@NAL based on gene models and supporting transcriptomic data on our newest *B. oleae* genome assembly (GCA_001188975.3). *Bo-Orco* spans a 14,416 bp genomic locus and consists of seven exons (open reading frame’s length is 1419 bp) that encode a protein of 473 amino acid residues (XP_014092453.1) (Fig. 1a).

**Fig. 2:**
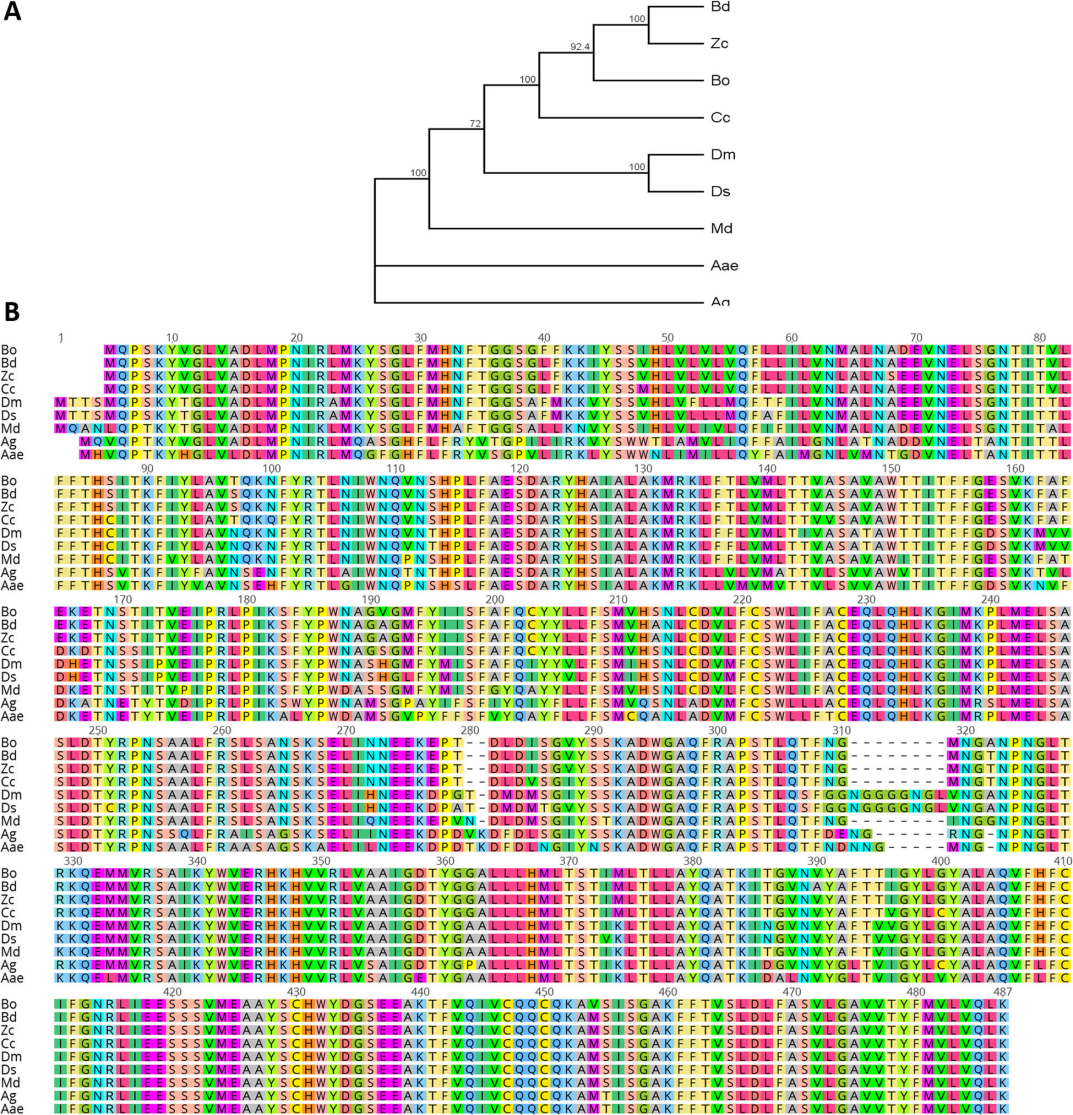
Comparison of Bo-Orco orthologs. **a** Phylogenetic analysis of Orco orthologs from different insect species. The distance tree was generated based on the NJ method, and bootstrap support values were generated based on 500 pseudoreplicates. **b** Alignment of the Bo-Orco amino acid residues to selected Orco proteins of various dipteran species. The abbreviation corresponds to the dipteran species: *Bactrocera oleae* (Bo); *Bactrocera dorsalis* (Bd); *Zeugodacus cucurbitae* (Zc); *Ceratitis capitata* (Cc); *Drosophila melanogaster* (Dm); *Drosophila suzukii* (Ds); *Anopheles gambiae* (Ag); *Aedes aegypti* (Aae); *Musca domestica* (Md)

Analysis of the membrane topology TMHMM prediction indicated that Bo-Orco has seven putative transmembrane α helical domains with the characteristic orientation of an intracellular N-terminus and an extracellular C-terminus (Fig. 1b). A multiple alignment analysis of the Bo-Orco protein with the predicted orthologues of various relative species across Diptera revealed high similarity, ranging from 75 to 98% (Fig. 2). Within the Tephritidae family, Bo-Orco shares almost 97% similarity, which is gradually reduced as evolutionary distance is increased, as also reported in other studies in tephritids [22, 23] and among insects in general [24]. This observation is consistent with the defined functional conservation of this co-receptor [24–26], indicating that *Bo-Orco* should also be of critical importance for the olive fruit fly’s olfaction. In addition, among the compared orthologous Orco genes, amino acid residues in the region of the C-terminus were highly conserved, in contrast with the specific residues between transmembrane domains that proved to be more variable (Fig. 2b).

### Expression profiles of *Bo-Orco*

**Fig. 3:**
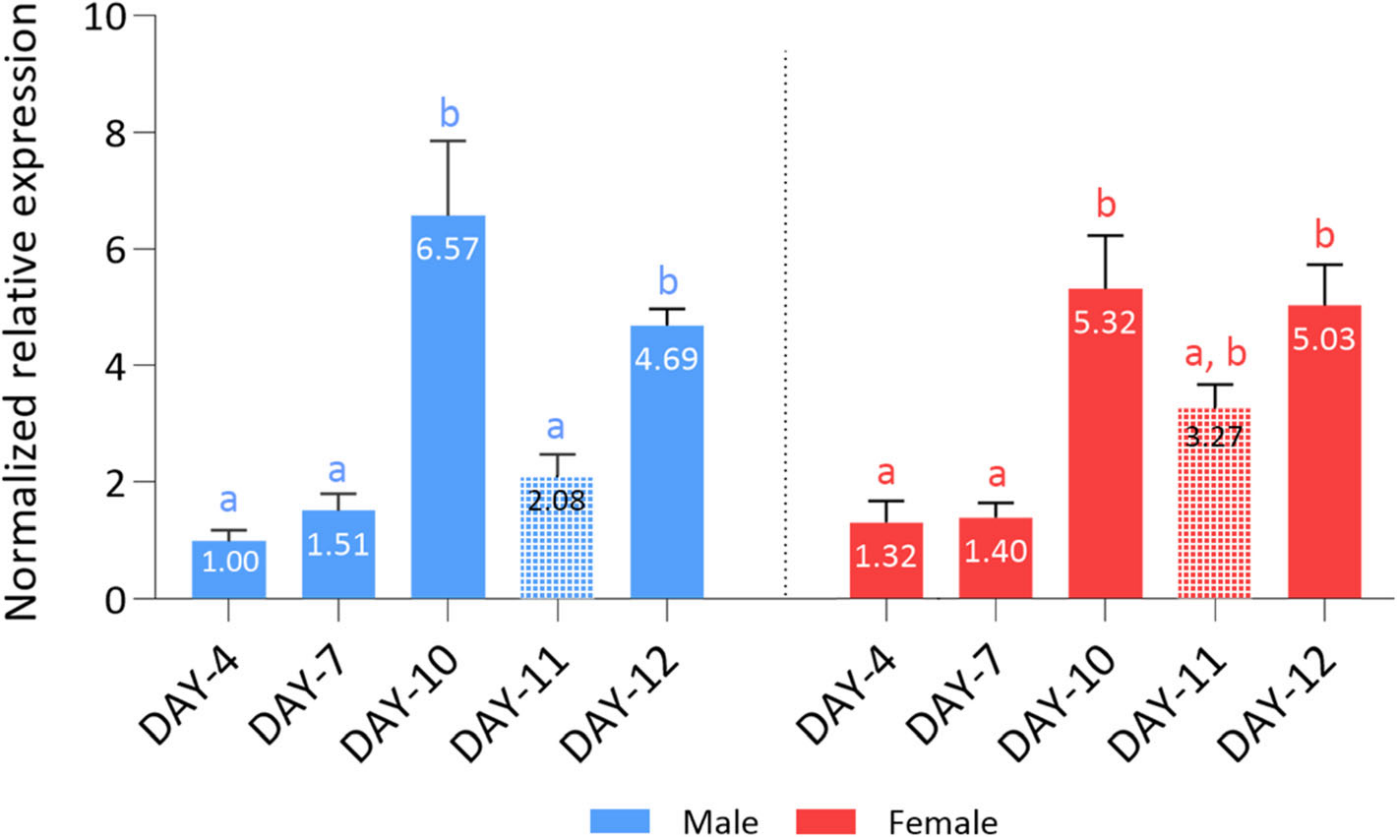
*Bo-Orco* expression profile in male and female antennae in pre- and post-mating communication. Relative transcription levels were measured based on their age (days after eclosion), sexual maturity, and mating status. The flies in DAY-4, − 7, − 10 and − 12 after eclosion were unmated and flies in DAY-11 correspond to flies after mating. Expression values were normalized using *Rpl19* and *actin3* as reference genes and shown relatively to male sample DAY-4. Data are presented as means ± Standard Deviation (SD). Small different letters next to the SD bars indicate a significant difference among different male or female samples respectively, in multiple comparisons within each sex group (*P* < 0.05, one-way ANOVA with Tukey’s test)

In order to investigate Orco’s differential gene expression relative to sexual maturity and mating status in both sexes, we determined the expression profile of *Bo-Orco* at different timepoints of adulthood between antennae of virgin and antennae of mated insects of both sexes separately. *Bo-Orco* relative expression was determined by qRT-PCR in the antennae of male and female heads under the examined physiological conditions. Results showed similar expression patterns between the two sexes: expression gradually increased during aging and peeked at DAY-10 after eclosion (Fig. 3). DAY-4 and DAY-7 flies were kept in separate rooms with no premating communication, therefore Orco expression was low. At DAY-10 and thereafter, flies were brought in close vicinity, eliciting premating communication. As a result, Orco expression increased statistically significantly (~ 5 fold for both sexes) compared to male sample DAY-4. On DAY-11, and after mating was concluded, Orco expression decreased. In males, this drop is significant and probably reflects males’ short term decreased receptivity to remating. Indeed, olive fruit flies undergo prolonged copulation (40 min to 2 h) at dusk [27], which limits the number of copulations to one per day, allowing males to replenish ejaculates for their possible copulation on the following evening [28]. Furthermore, in contrast to most tephritid fruit flies, the attractant pheromone is produced mainly by female flies [29]. Given the well-documented post-mating inhibition of remating in females of most species, including Tephritids [30–35], the drop of Orco expression in male olive fruit flies is more than anticipated. In females, on the other hand, the drop in Orco expression was intermediate, probably reflecting the fact that despite the short-term decrease receptivity in remating, Orco is also involved in the search of oviposition sites which would counterbalance the overall reduction of Orco expression.

### RNAi of *Bo-Orco* to determine its function

**Fig. 4:**
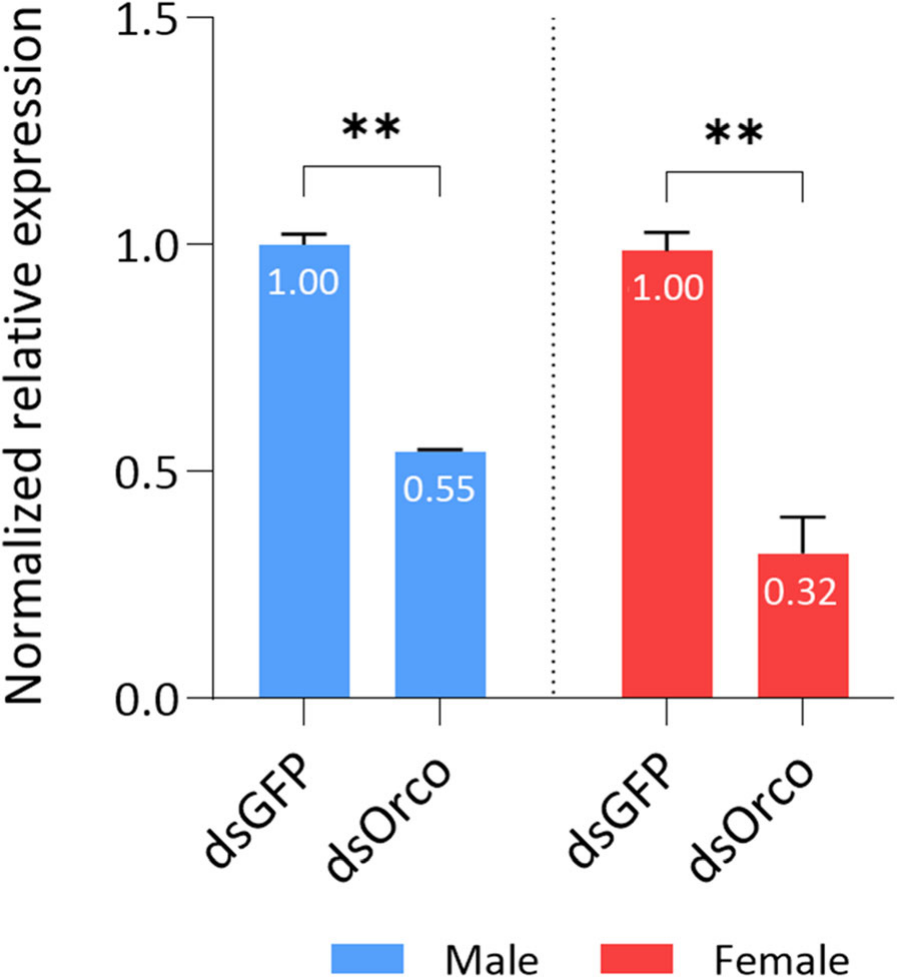
Relative quantitative analysis of *Bo-Orco* in the antennae of dsRNA injected *B. oleae* males and females. Expression levels were evaluated 72 h after injection and are shown relatively to control treatment, injected with dsGFP. All values were normalized using *Rpl19* and *actin3* as reference genes. Data are presented as means ± standard deviation (SD). Asterisks indicate the significant difference between the two samples per sex (*P* < 0.05, unpaired Student’s t-test)

The functional role of *Bo-Orco* was examined by RNAi silencing, selecting the dsRNA injections as the preferable way of delivery in sexually mature DAY-7 insects. Apart from the target gene, the *GFP* gene was also targeted as a control to assess both the experimental handling of the dsRNA and the possible effects on the insect’s physiological status by the activation of the RNAi mechanism. The efficiency of the RNAi treatment was evaluated by relative qRT-PCR (Fig. 4) in the insect’s antennae 3 days after injections.

As expected, dsGFP injections had no impact on insect physiology (no mortality was observed), nor on expression levels of *Bo-Orco.* Expression of *Bo-Orco* in the dsOrco-injected flies compared with the dsGFP-injected ones, was decreased in both sexes (Fig. 4). Specifically, a down regulation of ~ 45% was observed in males and ~ 68% in females respectively. The induced reduction in *Bo-Orco* transcription profile and the persistence of the gene silencing were sufficient evidence to proceed further with behavioral RNAi assays.

### Knockdown of *Bo-Orco* impairs mate recognition and oviposition

**Fig. 5:**
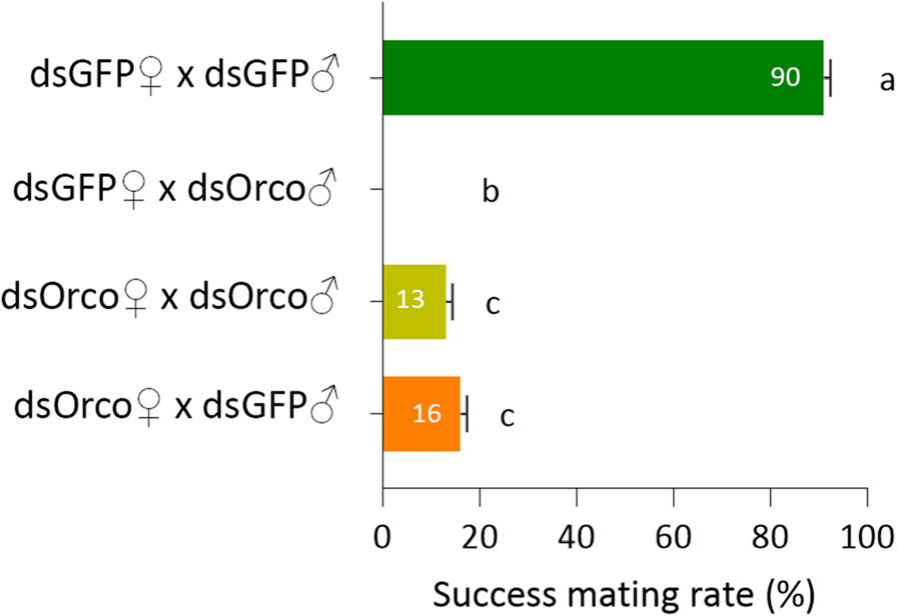
RNAi effect on mating behavior. Bars indicate the percentage of the successful matings between *Bo-Orco*-injected and/or control insects (dsGFP-injected) in all possible combinations. Data are presented as means ± standard deviation (SD). Small letters next to the SD bars indicate a significant difference among different samples (*P* < 0.05, one-way ANOVA with Tukey’s test)

To investigate the impact of Orco down-regulation on mate seeking, on DAY-7 after eclosion we injected sexually mature virgin males and females with dsRNA, and 3 days later we grouped them in all possible combinations, to generate putative mating pairs (Fig. 5). DsGFP-injected male and female flies in control group-i were able to mate successfully at high frequencies (90%). This result was expected given that the Orco protein was not affected and could elicit a normal behavioral response to sexual signals. Interestingly, in group-ii (dsGFP♀ & dsOrco♂) no copulation attempts were observed. This finding agrees with the proposed role of Orco in insects, as a necessary subunit to form a functional OR complex [26] and respond to chemical signals, in this case the sexual pheromone. Possibly *Bo-Orco* silencing suppressed male response to the female-emitted olean pheromone during the calling phase. However, this distinct phenotype of complete inability to mate, was not observed in group-iii (dsOrco♀ & dsOrco♂), in which also dsOrco♂ were tested and 13% of matings were confirmed. This finding can be attributed to the partial knockdown, in which a low amount of transcripts can still produce smaller amounts of a functional protein and therefore elicit a reduced behavioral response. Interestingly group-iv (dsOrco♀ & dsGFP♂), in which *Orco* was silenced only in females, appeared to have a considerable reduction in their ability to recognize their mates, since only ~ 16% of successful matings were observed. Therefore, *Orco* silencing in females, may also affect the process of courtship by reducing the female perception of the male pheromone muscalure during the contact phase.

**Fig. 6:**
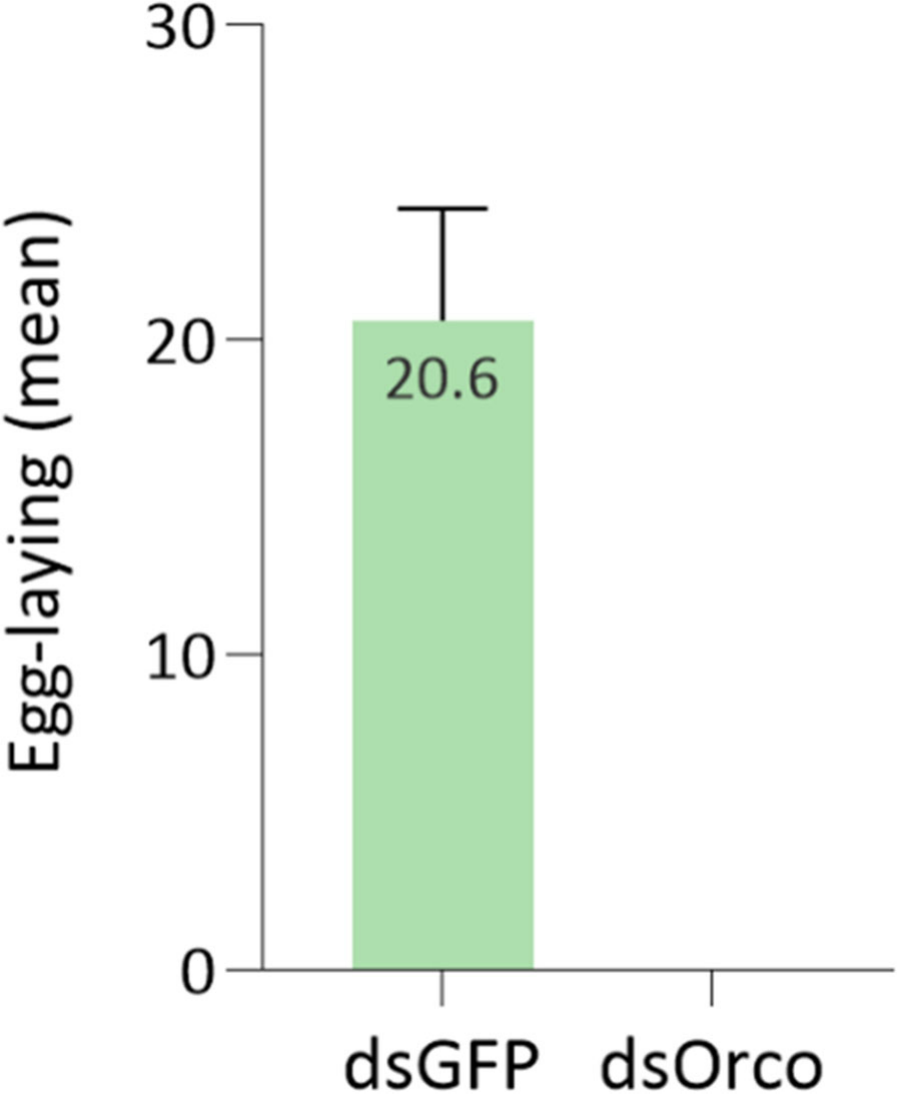
RNAi effect on oviposition of *Bo-Orco*-injected females, after mating once with wild type males (lab strain). The bar indicates the mean number of eggs laid per day (in biological duplicates) in individual cages during a period of 15 days after mating. Data are presented as means ± standard deviation (SD). Asterisks indicate the significant difference between the two samples (*P* < 0.05, unpaired Student’s t-test)

We further examined the effect of dsRNA-mediated *Orco* knockdown in *B. oleae* oviposition. In this experimental design, sexually mature virgin females were injected with dsOrco (1 μg/μl) and subsequently mated with wild type males, aiming to test whether the low percentage of the dsRNA female flies that can still mate, are also able to oviposit. *Orco*-silenced females that were visually observed to mate, were further daily examined regarding their ability to lay eggs. Interestingly, no eggs were collected during a 15-day period of observation. As a control, dsGFP injected virgin females were mated with wild type males; females laid an average of 20 eggs per day with a 100% hatching rate (Fig. 6). This result indicates a possible role of Orco either in the post-mating switch to induce post-mating responses or in the ability to recognize the oviposition substrate. However, both these hypotheses need further study in order to demonstrate the exact mechanism of Orco implication in the olfactory-mediated pathway of oviposition behavior. Camera recording could clarify whether flies made any attempts to find oviposition sites or they did not attempt at all. The activation of olfactory sensory neurons that express specific ORs can evoke egg-laying based on sensory cues, a process that might be inhibited by Orco knockdown. However, even mating-derived cues can promote such behaviors by engaging either chemosensation or mechanosensation [36]. In the first case, sperm transfer is the primary molecular trigger to be delivered by the male to the female. Therefore, in anosmic flies it should be further examined whether sperm was successfully delivered and if so, to examine if those flies respond to sperm presence by evoking the oviposition process. However, while the observation of oviposition reduction in Orco knocked-down flies was unambiguous, the elucidation of the molecular mechanistic details goes beyond the scope of the current analysis.

## Conclusion

Taken together, our analysis demonstrates that *Bo-Orco* is implicated in the olfactory reception of the olive fruit fly. RNAi silencing of *Orco* led to transient suppression of its expression which proved an effective approach to evaluate the behavioral effects in two distinct and fundamental physiological processes, mating and oviposition. Although partial, the knockdown of *Orco* expression in both males and females affected courtship and reduced mating ability. Additionally, *Orco* silencing led to complete egg-laying inhibition. This RNAi-induced effect on reproduction related processes indicates Orco’s crucial role in fly’s propagation. However, whether this oviposition inhibition is due to a consequent loss of the fly’s capacity to evoke oviposition or to inability to identify its oviposition substrate remains to be determined.

Overall, Orco could serve as a potential target for the disruption of the olfactory-driven reproductive behavior. Manipulating Orco would affect pre- and/or post- mating processes, ultimately reducing the insect’s reproductive success. Screening and deorphanization of chemoreceptors, albeit a challenging task, would allow the discovery of novel species-specific odorant-OR interactions that could also be directed towards the development of olfactory-based insect manipulation technologies.

## Materials and methods

### Insect samples

The *B. oleae* population used in the experimental procedure originated from the laboratory strain “Demokritos” Nuclear Research Centre, Athens, Greece. This strain has been maintained in our laboratory for over 15 years under controlled rearing conditions (25 ± 1 °C, 55 ± 5% RH) in a 16:8 h light:dark photoperiod. Both larvae and adults were reared on standard artificial diets and paraffin cones were used as oviposition substrates [37]. Upon adult emergence, female and male flies were separated and stored in different cages based on age until testing phase.

### *Bo-Orco* gene identification and sequence analysis

The *Bo-Orco* gene was identified by BLASTP homology analysis against the NCBI non-redundant (nr) protein database of *Bactrocera oleae* (taxid:104688) using the *D. melanogaster* orthologue as a query. Further manual curation of the gene structure was performed at the *B. oleae* genomic database (https://i5k.nal.usda.gov/Bactrocera_oleae) on the Apollo platform of the i5k Workspace@NAL [38]. Multiple sequence alignments were performed with Geneious® 10.2.6 (Biomatters Ltd.) based on ClustalW using the complete amino acid sequences retrieved from the GenBank under the following accession number: *Bactrocera dorsalis* (Bd-Orco, ACC86853.1); *Zeugodacus cucurbitae* (Zc-Orco, ADK97803.1); *Ceratitis capitata* (Cc-Orco, AAX14775.1); *Drosophila melanogaster* (Dm-Or83b, AAT71306.1); *Anopheles gambiae* (Ag-Orco, AAX14774.1); *Aedes aegypti* (Aae-Orco, NP_001345400.1); *Musca domestica* (Md-Orco, AFH96944.1). The transmembrane domains of Bo-Orco were analyzed using the TMHMM program (http://www.cbs.dtu.dk/services/TMHMM) and the topology was visualized using Protter [39]. The phylogenetic tree was constructed in Geneious® 10.2.6 (Biomatters Ltd.) using the neighbor-joining method (NJ) with 500 bootstrap pseudoreplications.

### RNA extraction, cDNA synthesis and relative quantitation expression analysis

Total RNA was extracted using TriSURE (Bioline) according to the manufacturer’s instructions, from antennae of female and male adult flies separately at different ages and physiological stages, as follows: i) DAY-4 after eclosion, sexual immaturity, ii) DAY-7 after eclosion, sexual maturity, iii) DAY-10 after eclosion, sexual maturity but unmated, iv) DAY-11 after eclosion, mated and v) DAY-12 after eclosion, sexual maturity but unmated. Males and females were kept in separate rooms to avoid premating communication. From DAY-10 and thereafter, cages were placed in close vicinity to allow premating communication. In each group 20 individuals were tested and each group was examined in two biological replicates.

cDNA synthesis and the subsequent relative quantitative real time PCR (qRT-PCR) was performed to analyze changes in expression levels of the selected genes as previously described [40]. Specific primers for the qRT-PCR amplification were designed for *Bo-Orco* by Primer-BLAST (http://www.ncbi.nlm.nih.gov/tools/primer-blast) (Table 1) to generate an amplicon of 90 bp length at an annealing temperature 56 °C. Gene expression values were normalized relatively to the housekeeping genes *Rpl19* and *actin3*, as suggested by Sagri et al. (2017). All amplifications were performed with two technical replicates. The reactions were carried out on Bio-Rad Real-Time thermal cycler CFX96 (Bio-Rad, Hercules, CA, USA) and relative gene expression data were analyzed using the CFX Manager™ software.

**Table 1:** Primers used for qRT-PCR and generating dsRNAs. Primer sequences for *RpL19* and *actin3* are described in [28]. Lowercase letters correspond to the T7 sequence

	Primer	Primer sequence (5’> 3′)
For qRT-PCR	c57709_g1_Orco_F3	CTTCTTTGGCGAGAGTGTGA
	c57709_g1_Orco_R3	GGCATTCCACGGATAGAAAGA
	*Rpl19*_F	AACAAACGTGTACTGATGG
	*Rpl19*_R	CACGTACTTTATGTCGTCTG
	*actin3_*F	CCACCAGAACGTAAATACTC
	*actin3_*R	TCTCATTGAGCGTTTAGAAG
For dsRNA synthesis	c57709_Orco_1_F	taatacgactcactatagggCAATGAGGAGAAGGAACCCA
	c57709_Orco_1_R	taatacgactcactatagggCAGTGAAGAACTTCGCTCCC
	GFP-F	taatacgactcactatagggCCGCCAGTGTGCTGGAA
	GFP-R	taatacgactcactatagggGATATCTGCAGAATTCGCC

### Synthesis of dsRNA

For the *Bo-Orco* dsRNA synthesis, a 545 bp PCR product was generated using primers containing the T7 RNA polymerase promoter (Table 1), designed by the ERNAi [41]. Similarly, a GFP-dsRNA was generated to be used as a control, based on the unrelated *GFP* gene (Green Fluorescent Protein). Each PCR product was further used as a template for in vitro transcription using the MEGAscript RNAi kit (Ambion, Austin, TX, USA) according to the manufacturer’s instructions.

### RNAi of *Bo*-*Orco* gene

#### dsRNA treatment

Male and female flies were separated by sex upon adult emergence and kept in different cages. A group of 100 flies of each sex (in biological duplicates) were selected randomly from the sexed populations on DAY-7 after eclosion and subjected to individual injections with the Orco-dsRNA (dsOrco) (1 μg/μl) diluted in ddH_2_O. Another group as described above but injected with GFP-dsRNA (dsGFP) (1 μg/μl) was used as a control. Each fly was injected with 69 nl of dsRNA at the metathoracic segment using the Nanojet II (Drummond, Broomall, PA, USA) and glass needles under a Leica stereoscope. The injected female and male flies were then placed separately by sex into small cages, according to the injected dsRNA, for further treatment, regarding either the evaluation of RNAi efficiency or the behavioral bioassays (see next section).

#### RNAi efficiency

The RNAi efficiency was evaluated based on the transcription levels of the silenced *Bo-Orco* gene, which were analyzed by qPCR (as described above). Each cDNA for the qPCRs was synthesized using the RNA extracted from pooled antennae samples according to the protocol described in the previous section. Each sample (in biological duplicates) consisted of 10 sexed individuals collected 3 days after injection (DAY-10 after eclosion).

### RNAi mating and oviposition assays

#### Mating assays

In mating assays we used the sexually mature virgin males and females that were injected with either dsOrco (1 μg/μl) or dsGFP (1 μg/μl) on DAY-7 after eclosion, as described above, and were kept separately in different cages based on their sex. Three days after dsRNA application, 10 males and 10 females were grouped in small cages, based on all possible combinations of injections. Groups were made as follows (in biological duplicates): i) dsGFP♀ & dsGFP♂, ii) dsGFP♀ & dsOrco♂, iii) dsOrco♀ & dsOrco♂, iv) dsOrco♀ & dsGFP♂. Mixed sex groups were further optically observed to confirm the successful mating and the single mating pairs were isolated in different cages until the mating procedure was completed (mating time > 30 min).

#### Oviposition assays

In oviposition assays we used the sexually mature virgin females that were injected with either dsOrco (1 μg/μl) or dsGFP (1 μg/μl) on the DAY-7 after eclosion. Subsequently they were transferred in different cages according to the application. Three days after dsRNA application, 10 injected females were put in small cages, and 10 wild type males were added in each cage as follows (in biological duplicates): i) dsGFP♀ & wild type♂, ii) dsOrco♀ & wild type♂. Mixed sex groups were further visually observed to confirm the successful mating and isolate the mating pairs (copulation > 30 min). Mated females were transferred to individual cages, with food and water supply and an oviposition cone. Each cone was washed with dH_2_0 and the collected eggs were measured under a stereomicroscope daily for a 15-days period. Eggs were further transferred to larval food to estimate the larval hatching rate as well.

### Statistical analysis

All data are expressed as means ± standard deviation (SD). When multiple comparisons were performed among different sample means, significant differences were analyzed using the one-way analysis of variance (ANOVA) and Tukey’s post-hoc test. In the case of simple comparisons between two means, significant differences were analyzed using the unpaired Student’s t-test. For both types of statistical analyses, a level of *p*-value< 0.05 was set as statistically significant. All statistical analyses were performed using the PRISM 8.0 software (GraphPad Software, San Diego, California, USA).

## Data Availability

The *B. oleae* sequences are accessible via the genome browser available at i5k Workspace@NAL: https://i5k.nal.usda.gov/Bactrocera_oleae. The sequences of the Orco orthologs used in this study are accessible in NCBI’s GenBank under the following accession numbers: *Bactrocera dorsalis* (ACC86853.1); *Zeugodacus cucurbitae* (ADK97803.1); *Ceratitis capitata* (AAX14775.1); *Drosophila melanogaster* (AAT71306.1); *Anopheles gambiae* (AAX14774.1); *Aedes aegypti* (NP_001345400.1); *Musca domestica* (AFH96944.1).

## Abbreviations

ORNs: Olfactory receptor neurons
OBPs: Odorant binding proteins
ORs: Olfactory receptors
Orco: Odorant receptor co-receptor
qRT-PCR: Relative quantitative real time PCR
dsRNA: Double strand RNA
RNAi: RNA interference
GFP: Green fluorescence protein
dsGFP: GFP-dsRNA
dsOrco: Orco-dsRNA

## References

[1] Katsoyannos P. Olive pests and their control in the near east, vol. 115: FAO plant production and protection paper; 1992.

[2] Tzanakakis ME (2006). Insects and mites feeding on olive: distribution, importance, habits, seasonal development, and dormancy: brill.

[3] Gadenne C, Barrozo RB, Anton S (2016). Plasticity in insect olfaction: to smell or not to smell?. Annu Rev Entomol.

[4] Leal WS (2013). Odorant reception in insects: roles of receptors, binding proteins, and degrading enzymes. Annu Rev Entomol.

[5] Jones WD, Nguyen T-AT, Kloss B, Lee KJ, Vosshall LB (2005). Functional conservation of an insect odorant receptor gene across 250 million years of evolution. Curr Biol.

[6] Wicher D, Schäfer R, Bauernfeind R, Stensmyr MC, Heller R, Heinemann SH, Hansson BS (2008). Drosophila odorant receptors are both ligand-gated and cyclic-nucleotide-activated cation channels. Nature.

[7] Sato K, Pellegrino M, Nakagawa T, Nakagawa T, Vosshall LB, Touhara K (2008). Insect olfactory receptors are heteromeric ligand-gated ion channels. Nature.

[8] Butterwick JA, del Mármol J, Kim KH, Kahlson MA, Rogow JA, Walz T, Ruta V (2018). Cryo-EM structure of the insect olfactory receptor Orco. Nature.

[9] Liscia A, Angioni P, Sacchetti P, Poddighe S, Granchietti A, Setzu MD, Belcari A (2013). Characterization of olfactory sensilla of the olive fly: behavioral and electrophysiological responses to volatile organic compounds from the host plant and bacterial filtrate. J Insect Physiol.

[10] Canale A, Germinara SG, Carpita A, Benelli G, Bonsignori G, Stefanini C, Raspi A, Rotundo G (2013). Behavioural and electrophysiological responses of the olive fruit fly, *Bactrocera oleae* (Rossi) (Diptera: Tephritidae), to male- and female-borne sex attractants. Chemoecology.

[11] Malheiro R, Ortiz A, Casal S, Baptista P, Pereira JA (2015). Electrophysiological response of *Bactrocera oleae* (Rossi)(Diptera: Tephritidae) adults to olive leaves essential oils from different cultivars and olive tree volatiles. Ind Crop Prod.

[12] Levi-Zada A, Nestel D, Fefer D, Nemni-Lavy E, Deloya-Kahane I, David M (2012). Analyzing diurnal and age-related pheromone emission of the olive fruit fly, *Bactrocera oleae* by sequential SPME-GCMS analysis. J Chem Ecol.

[13] Benelli G, Canale A, Bonsignori G, Ragni G, Stefanini C, Raspi A (2012). Male wing vibration in the mating behavior of the olive fruit fly *Bactrocera oleae* (Rossi)(Diptera: Tephritidae). J Insect Behav.

[14] Gerofotis CD, Ioannou CS, Papadopoulos NT (2013). Aromatized to find mates: α-pinene aroma boosts the mating success of adult olive fruit flies. PLoS One.

[15] Mazomenos B, Haniotakis G (1981). A multicomponent female sex pheromone of *Dacus oleae* Gmelin: isolation and bioassay. J Chem Ecol.

[16] Mazomenos B, Haniotakis G (1985). Male olive fruit fly attraction to synthetic sex pheromone components in laboratory and field tests. J Chem Ecol.

[17] Baker R, Herbert R, Howse PE, Jones OT, Francke W, Reith W. Identification and synthesis of the major sex pheromone of the olive fly (*Dacus oleae*). J Chem Soc Chem Commun. 1980;(2):52–3.

[18] Carpita A, Canale A, Raffaelli A, Saba A, Benelli G (2012). Raspi a: (Z)-9-tricosene identified in rectal gland extracts of *Bactrocera oleae* males: first evidence of a male-produced female attractant in olive fruit fly. Naturwissenschaften.

[19] Benelli G, Bonsignori G, Stefanini C, Raspi A, Canale A (2013). The production of female sex pheromone in *Bactrocera oleae* (Rossi) young males does not influence their mating chances. Entomol Sci.

[20] Lagos D, Koukidou M, Savakis C, Komitopoulou K (2007). The transformer gene in *Bactrocera oleae*: the genetic switch that determines its sex fate. Insect Mol Biol.

[21] Gregoriou ME, Mathiopoulos KD (2020). Knocking down the sex peptide receptor by dsRNA feeding results in reduced oviposition rate in olive fruit flies. Arch Insect Biochem Physiol.

[22] Zheng W, Zhu C, Peng T, Zhang H (2012). Odorant receptor co-receptor Orco is upregulated by methyl eugenol in male *Bactrocera dorsalis* (Diptera: Tephritidae). J Insect Physiol.

[23] Yi X, Zhao H, Wang P, Hu M, Zhong G (2014). Bdor⧹ Orco is important for oviposition-deterring behavior induced by both the volatile and non-volatile repellents in *Bactrocera dorsalis* (Diptera: Tephritidae). J Insect Physiol.

[24] Missbach C, Dweck HK, Vogel H, Vilcinskas A, Stensmyr MC, Hansson BS, Grosse-Wilde E (2014). Evolution of insect olfactory receptors. Elife.

[25] Stengl M, Funk NW (2013). The role of the coreceptor Orco in insect olfactory transduction. J Comp Physiol A.

[26] Benton R, Sachse S, Michnick SW, Vosshall LB (2006). Atypical membrane topology and heteromeric function of Drosophila odorant receptors in vivo. PLoS Biol.

[27] Mazomenos B (1989). Dacus oleae. World crop pests.

[28] Gerofotis C, Yuval B, Ioannou C, Nakas C, Papadopoulos N (2015). Polygyny in the olive fly—effects on male and female fitness. Behav Ecol Sociobiol.

[29] Haniotakis G (1974). Sexual attraction in the olive fruit fly, *Dacus oleae* (Gmelin). Environ Entomol.

[30] Manning A (1962). A sperm factor affecting the receptivity of *Drosophila melanogaster* females. Nature.

[31] Chen PS, Stumm-Zollinger E, Aigaki T, Balmer J, Bienz M, Böhlen P (1988). A male accessory gland peptide that regulates reproductive behavior of female D. melanogaster. Cell.

[32] Chapman T, Bangham J, Vinti G, Seifried B, Lung O, Wolfner MF, Smith HK, Partridge L (2003). The sex peptide of *Drosophila melanogaster*: female post-mating responses analyzed by using RNA interference. Proc Natl Acad Sci.

[33] Yapici N, Kim Y-J, Ribeiro C, Dickson BJ (2008). A receptor that mediates the post-mating switch in Drosophila reproductive behaviour. Nature.

[34] Nakagawa S, Farias G, Suda D, Cunningham R, Chambers D (1971). Reproduction of the Mediterranean fruit fly: frequency of mating in the laboratory. Ann Entomol Soc Am.

[35] Delrio G, Cavalloro R (2016). Influenza dell'accoppiamento sulla recettività sessuale e sull'ovideposizione in femmine di *Ceratitis capitata* Wiedemann. Entomologica.

[36] Cury KM, Prud’homme B, Gompel N (2019). A short guide to insect oviposition: when, where and how to lay an egg. J Neurogenet.

[37] Tzanakakis M (1989). Small scale rearing: *Dacus oleae*. Fruit Flies.

[38] Poelchau M, Childers C, Moore G, Tsavatapalli V, Evans J, Lee C-Y, Lin H, Lin J-W, Hackett K (2014). The i5k workspace@ NAL—enabling genomic data access, visualization and curation of arthropod genomes. Nucleic Acids Res.

[39] Omasits U, Ahrens CH, Müller S, Wollscheid B (2013). Protter: interactive protein feature visualization and integration with experimental proteomic data. Bioinformatics.

[40] Sagri E, Koskinioti P, Gregoriou M-E, Tsoumani KT, Bassiakos YC, Mathiopoulos KD (2017). Housekeeping in Tephritid insects: the best gene choice for expression analyses in the medfly and the olive fly. Sci Rep.

[41] Horn T, Boutros M (2010). E-RNAi: a web application for the multi-species design of RNAi reagents—2010 update. Nucleic Acids Res.

